# Indents

**Published:** 1918-04

**Authors:** 


### INDENTS

**☞ Brief Articles of Technical or Scientific Value will receive notice and comment. Contributions invited.**

*Advice to the Dentist's Assistant*

I may be told that initiative is heaven-born and inherent—that it can not be acquired. Bosh! Let me tell you what is necessary. Thinking is necessary, concentrating is necessary, observing is necessary. If you have the interests of the office at heart you will think often and seriously as to means of bettering your service, you will concentrate your mind on the affairs of the office, you will be observant at all times of the little amenities of life that go so far toward making matters move smoothly. This is initiative. It is this which makes the young lady assistant indispensable in the conduct of a practice, which binds her to her employer, and makes their interests mutual.

But you can not acquire this kind of initiative unless your mind is on your work, unless you take a real interest in it. If you are working solely for the salary you receive, or if your mind is more on dances than on dentistry, you will never be anything else than a routinist, and mostly a poor one that. Nothing of value was ever obtained without application, and no walk of life was ever properly graced by an individual without enthusiastic interest in the work in hand. If you can not concentrate on your duties so as to serve the best interests of your employer, then in justice to him and yourself leave him, and do something else. Maybe you were meant to be an expert dancer.

By all of this I do not mean that you should have no pleasures or diversions. I honestly want to see you have both. No human being should be condemned to a life of monotony or drudgery—least of all a young lady with the qualities necessary to assist in a dental office. But here are two things worthy of consideration—first that there are many diversions other than those which incapacitate you for your work; and second, that if you perform your duties conscientiously and capably you will find much real diversion in the work itself. There is no solace quite so concrete as the satisfaction of having excelled in any service, and I commend this to your most careful consideration.—C. N. Johnson, in *Review.*

*Slow Injections* Sufficient time should always be allowed in order to give the tissue in the immediate vicinity of the needle point ample time to adjust itself to the new order of things. If the solution is injected too rapidly the patient in most cases will encounter pain, not only at the time of the injection, but a slight amount of trauma is produced, which produces postoperative pain. Nerve blocking injections can be made with practically no pain to the patient if the operator is skillful with his technic. Those of you who have experienced nerve blocking injections I am quite sure appreciated the ease of application by the slow injection of the solution.—A. E. Smith, in *Review.*

*Teeth Do Not Control Mandibular Movement* When you are building up cusps and carving fissures it will be well to remember that the teeth do not control the mandibular movements. If a cusp or part of a crown is not in harmony with the arcs through which the mandible sweeps in its movements, it will wear off, or act as a lever to loosen the tooth upon which it is found. When several teeth are malposed they will to some extent limit the movements of the mandible, but never control it. The mandible will move as freely and as far as the muscles will pull, or the patient chooses to move it, but in all these movements, it sweeps through certain arcs of circles that are in harmony with the peculiarity of the anatomics of the case, without any regard to the occlusion or the occlusal surfaces of the units that make up the two arches. If any cusp or series of cusps are so placed that they interfere with these movements, they will be reduced to a point where they are in harmony or the tooth of which they are a part will be exfoliated, while the mandible swings merrily on in its accustomed arcs.—F. W. Frahm, in *Pacific Gazette.*

*Conductive Anesthesia* Don't attempt to carry on conductive anesthesia until you are properly trained to do so. It is well to read and become familiar with the literature and see others use it or demonstrate it before attempting it yourself. That will give you a good preliminary idea of what can be done, but if you practice it without being instructed by men of experience your results will be unsatisfactory. Conductive anesthesia, when properly administered, is one of the greatest boons that science and invention have ever brought to the doctor and patient.—J. E. Nyman, in *Review.*

*Clinical Methods of Treating Hypersensitive Dentin* A necessary prerequisite to any rational method of treating hypersensitive dentin is a consideration of the factors, structures, or conditions which permit of this hypersensitiveness.

The entire question of a nerve supply to the dentin is still being studied. Prinz suggests that if there are nerve fibers in the dentin, they are not of a sensory or motor type, but are trophic in function.

However, this question may be decided in the future, for the present Prinz advances a simple adequate explanation of the mechanism of hypersensitivity. The basis of this theory is the practical incompressibility of water. The sequence of events as Prinz pictures them is somewhat as follows:

There is a break in the enamel covering. The thereby exposed fibrillæ of the dentin absorb water from the saliva and swell; then the slightest pressure upon this swollen dentin will be transmitted through the odontoblastic processes to the bodies of the odontoblasts, and these in turn will compress the nerve plexus underlying and encircling the odontoblasts.

The validity of this explanation is supported by the clinical experience that dehydration will remedy hypersensitiveness. The warm air blast, alcohol, and acetone will dehydrate dentin, but penetrate so short a distance that frequent repetition is necessary. Sharp instruments used with dexterity conduce to minimize the pressure exerted upon the swollen dentin.

The use of agents which anesthetize the tooth by lowering its temperature is unsatisfactory.

Two classes of drugs may be used to overcome the hypersensitivity—caustics and anesthetics. Among the caustics are mercuric chlorid, silver nitrate, zinc chlorid, sodium or potassium hydroxid, and phenol. All these agents act only superficially, and their application must be continually repeated. Consequently, except in slight cases of hypersensitivity, they are rather unsatisfactory.

The condition of analgesia induced by a general anesthetic, *e. g.* nitrous oxid, is not completely satisfactory. So deep an anesthesia that all sensation is inhibited alone meets the requirements of many cases.

Prinz's views upon local anesthesia and the technic of its induction are so well known and so accessible that they need not be given here.

In closing, he emphasizes the danger and needlessness of resorting to copyrighted preparations used for desensitizing purposes. The employment of preparations containing arsenic or formaldehyd is pleasantly satirized. Extract from *Dental Outlook* by *Dental Cosmos.*

*Crown and Bridge Investment* A very serviceable investment may be made by mixing plaster of paris and hard coal ashes. The ashes are prepared by sifting through a fine sieve until all grit is removed and only the fine, soft powder remains. Take one part of the ashes and two parts of the plaster and sift them together very carefully. A small amount of Venetian red may be added to assist in ascertaining whether the mix is uniform. This investment will not warp or crack under the blow-pipe heat, and is superior to many of the patented compounds.—F. W. Frahm, in *Pacific Gazette.*

*A Real Bull'* From a speech by the Lord Mayor of Dublin: "That would be a crying evil, to leave the poor people in the city without milk. It would be a wise thing if the Corporation would take the bull by the horns and deal with the matter."—*Dublin Evening Mail.*

*Harelip and Clelt Palate* New says that in the Mayo Clinic they prefer to close the lip first when the child is between three and four months old, if he is gaining weight and doing well. From three or four days to a week before the operation the child should be fed with a spoon or dropper to accustom it to this method of feeding, since of course, after the operation, it is not allowed to nurse from the bottle or the breast. When there is a cleft of the alveolar process, as in the complete single harelip, the lip is brought together over it, but no attempt is made to approximate the alveolar process. The same procedure is used in the treatment of the premaxilla. In a case of double harelip the lip is brought together over the premaxilla and its normal rounded appearance is maintained. If the alveolar process is forced back in the single harelip or if the premaxilla is removed in the double harelip or a wedge-shaped piece is taken out of the vomer and the premaxilla forced back, the lip will be flattened and it will be almost impossible to correct the deformity. When united, the lip gradually presses back the alveolar process or premaxilla into its normal position, giving the normal rounded contour to the face and the correct alignment to the teeth. The time for closing the palate is when the child is from a year to a year and a half old, before it has begun to talk. The edge-to-edge approximation or the Langenbeck operation is the operation of choice for the closure of cleft palate.—*Minnesota Medicine.*

*He Got Ten Years* A negro who was well known to the judge had been haled into court on a charge of having struck a relative with a brick. After the usual preliminaries the court inquired:

"Why did you hit this man?"

"Jedge, he called me a black rascal."

"Well, you are one, aren't you?"

"Yessah, maybe I is one. But, jedge, s'pose some one should call you a black rascal, wouldn't you hit 'em?"

"But I'm not one, am I?"

"Naw sah, naw, sah, you ain't one; but s'pose some one'd call you de kind of rascal you *is,* what'd you do?"

*Plate Polishing* Metal plates will take on a very high lustre when a little aqua ammonia is used in mixing the chalk instead of water. Vulcanite will take on a mirror polish if the finishing is done by the use of tin oxid made into a paste with glycerin. In either case a clean buff should be chosen and kept exclusively for this purpose.—F. W. Frahm, in *Pacific Gazette.*

*The Violet Ray* One of the most valuable claims advanced for the use of the violet ray is its power to sterilize the part adjacent to the driving point. Here is Dr. Reed's explanation of this property: "The tissues and blood contain a large amount of sodium chloride, the normal saline solution. When a charge of electricity is passed through a wet medium, electrolysis takes place and the sodium chloride is changed into sodium hypochlorite, a powerful oxydizing agent and germicide, the action of which on tissues sterilizes the part. In this chemical change there are several intermediate changes which take place and subsidiary products are formed, but the final result is the production temporarily of sodium hyperchlorite, the sterilizing properties of which accomplish the result desired. It is not instantaneous sterilization, and the time of application has much to do with its permanency. Its value in this respect has been demonstrated by the cure of chronic abscesses without root amputation." The application of the violet ray may be of use in reducing a swelling, and its value in this connection lies in the fact that the molecular bombardment of the tissues produced by its application starts up and diffuses the congested circulation which has caused the swelling. "The high frequency generator produces an electric current that increases the body heat without increasing the temperature. It stimulates by the increased production of oxygen in the form of ozone and by the increased force and volume of the chemically molecular energy, has a highly energizing effect locally and produces an increased tonicity of the part adjacent to the point of application."—*Oral Health.*

*Infiltration Anesthesia* In anesthetizing the upper bicuspids for cavity preparation or pulp removal or for extraction of teeth, I have made it a practice to insert the needle near the apex of the root, where the buccal mucosa is reflected from the alveolar process, inserting the needle with the beveled side in, next to the process and right under the periosteum, making a subperiosteal injection. I have had no difficulty with that type of injection. The places where I have had difficulty with infiltrative anesthesia has been on lower molars and bicuspids. I do not get complete anesthesia here unless I use the conductive type of injection, 2 c. c. of a two per cent solution of novocain, in the region of the mandibular foramen.—Dr. Puterbaugh, in *Review*.

*War and Dental Supply Business* It may be interesting to know what the European war has cost the dental supply houses of Europe. Some idea may be had by the effect on the business of the Ash & Sons Co., of London.

Over four hundred of the company's employes have served or are serving with the British army.

The Central Powers have confiscated, sold out, and appropriated the company's branch houses in Brussels, Berlin, Munich, Breslau, Frankfurt, Hamburg, Vienna, Budapest, Lemberg, Bucharest and Constantinople, placing the proceeds in their respective Imperial Treasuries, the loss to the company being over one million dollars.—*Oral Health*.

*Mustard-oil Inhalation for Toothache* A recent writer in the *Muenchener Medizinische Wochenschrift* makes known what he pronounces a prompt cure for toothache, this consisting simply in shutting the eyes tight and then sniffing good and well upon a small vial containing volatile oil of mustard. The relief is asserted to be almost instantaneous, after the certainly very unpleasant byeffects have passed away. Also, the excruciating pain caused by inflammation of the middle ear is stopped by a single such inhalation. Of course, the relief is merely temporary, helpful though, until proper curative measures can be carried out.

Without wishing to cast doubt upon this report, the Abstractor is reminded of a little anecdote told by his father half a century ago, as a takeoff on Homeopathy. A wealthy Englishman visiting in Paris called upon a fashionable Homeopathist to get cured of an obstinate facial neuralgia. The great doctor held a little vial under his nose, bade him to smell upon it, then pompously announced: "You are cured!" Asked for his fee, the doctor said, "Ten louisdore." Thereupon the unconvinced Britisher pushed his purse under the doctor's nose and said: "Smell! You are paid."—*Clinical Medicine.*

*To Prevent Alcoholic Shock to the Pulp* The pulp of a tooth receives frequent shock from the careless use of alcohol in the tooth cavity. Alcohol is a marked refrigerant when it is used cold, for this reason I have found it a good technic to ignite for a second or two the alcohol moistened cotton and extinguish it before placing it in the cavity. The results are very gratifying to the patient and in no way defeats our purposes.—Dr. Frahm, in *Pacific Gazette.*

*Sweating Feet* I desire to add my experience, which has proved, by more than thirty years' trial, that ordinary powdered alum is a specific for sweating of the feet. For one who has suffered subjectively or objectively from perspiring or odorous feet, any decided relief is a great boon. Such relief does not come from bathing. It is not a matter of cleanliness alone. If the patient will simply dust the feet with powdered alum, also dusting some of it into stocking or sock before putting the stocking or sock on the foot, and will then dust some more of the powder into the shoe before putting it on, and will keep water off the feet, the relief will be prompt and most gratifying.—Dr. Reichard, in *Journal A. M. A.*

*Post Operative Pain from Extracting a Tooth* Almost immediate relief may be given the patient by inserting a pellet of cotton wet with chloroform to the full depth of the root socket and place your finger firmly over the mouth of the socket for from ten to twenty seconds, then remove the cotton from the socket. Repeat if necessary.—H. A. Cross, in *Review.*

